# Supplementary material for: Abstinence from Escalation of Cocaine Intake Changes the microRNA Landscape in the Cortico-Accumbal Pathway
Source: Biomedicines. 2023 May 5;11(5):1368. doi: 10.3390/biomedicines11051368 (PMC10216163; doi:10.3390/biomedicines11051368)
Supplement: Supplementary file 1 [file biomedicines-11-01368-s001.zip › biomedicines-2320424-supplementary final.pdf]

Abstinence from escalation of cocaine intake changes the microRNA landscape in the cortico-accumbal pathway

(Supplementary Materials)

Figure S1. Dissection of rat brain nucleus accumbens and prefrontal cortex.

Figure S2. Cocaine withdrawal/abstinence-associated miRNA-mRNA-pathway network predicted by IPA.

Figure S3. IPA miRNA Target Filter prediction of transcription factors (TFs) & nuclear receptors (NRs) potentially targeted by differentially expressed miRNAs in the IL-PFC of rats with protracted abstinence.

Figure S4. IPA miRNA Target Filter prediction of miRNA-mRNA-pathway regulatory networks containing miRNAs differentially expressed in the IL-PFC of rats with protracted abstinence.

Figure S5. IPA miRNA Target Filter prediction of miRNA-mRNA-pathway regulatory networks containing miRNAs differentially expressed in the PL-PFC of rats with either withdrawal or protracted abstinence.

Table S1. The concentration and RNA integrity number (RIN) of 36 rat brain RNA samples

Table S2. Addiction behavior Z scores and addiction index of rats with an 18-hr withdrawal or a 4-week abstinence

Table S3a. Correlation of addiction index with expression levels of top differentially expressed miRNAs ($|FC| \geq 2.0$ & $P < 0.05$) in three rat brain regions by partial correlation analysis

Table S3b. Correlation of Z ESC with expression levels of top differentially expressed miRNAs ($|FC| \geq 2.0$ & $P < 0.05$) in three rat brain regions by partial correlation analysis

Table S3c. Correlation of Z PR with expression levels of top differentially expressed miRNAs ($|FC| \geq 2.0$ & $P < 0.05$) in three rat brain regions by partial correlation analysis

Table S3d. Correlation of Z Shock with expression levels of top differentially expressed miRNAs ($|FC| \geq 2.0$ & $P < 0.05$) in three rat brain regions by partial correlation analysis

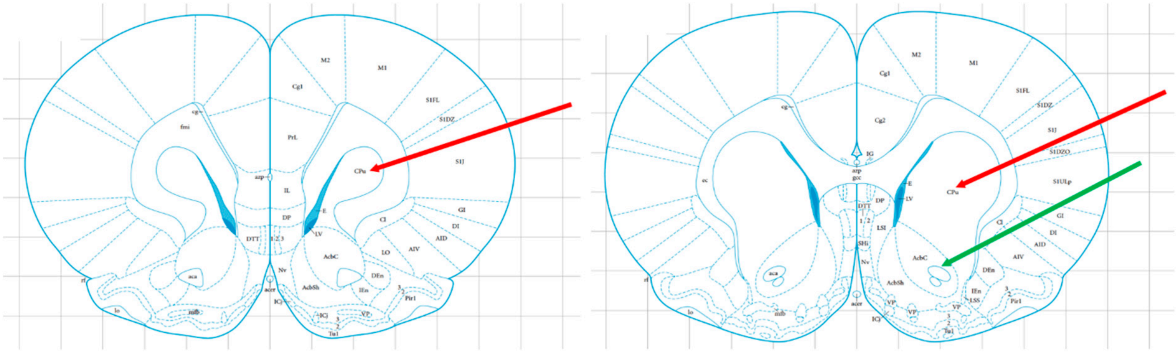

Figure S1a: Rat nucleus accumbens

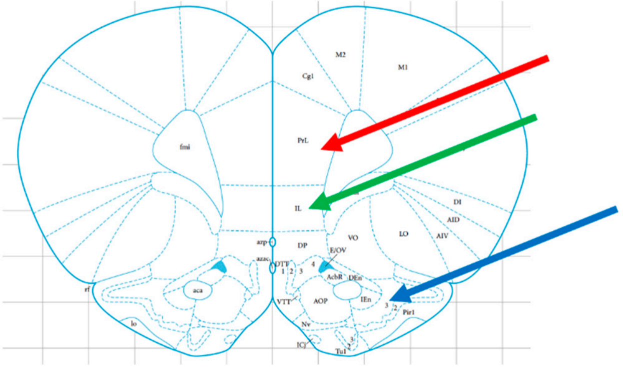

Figure S1b: Rat prefrontal cortex

Figure S1. Dissection of rat brain nucleus accumbens and prefrontal cortex. Figure S1a: The red arrow indicates the striatum [or corpus striatum (CPu)] and the green arrow indicates the anterior commissure. Figure S1b: The red arrow indicates prelimbic prefrontal cortex; the green arrow indicates infralimbic prefrontal cortex; and the blue arrow indicates the recognizable ventral portion.

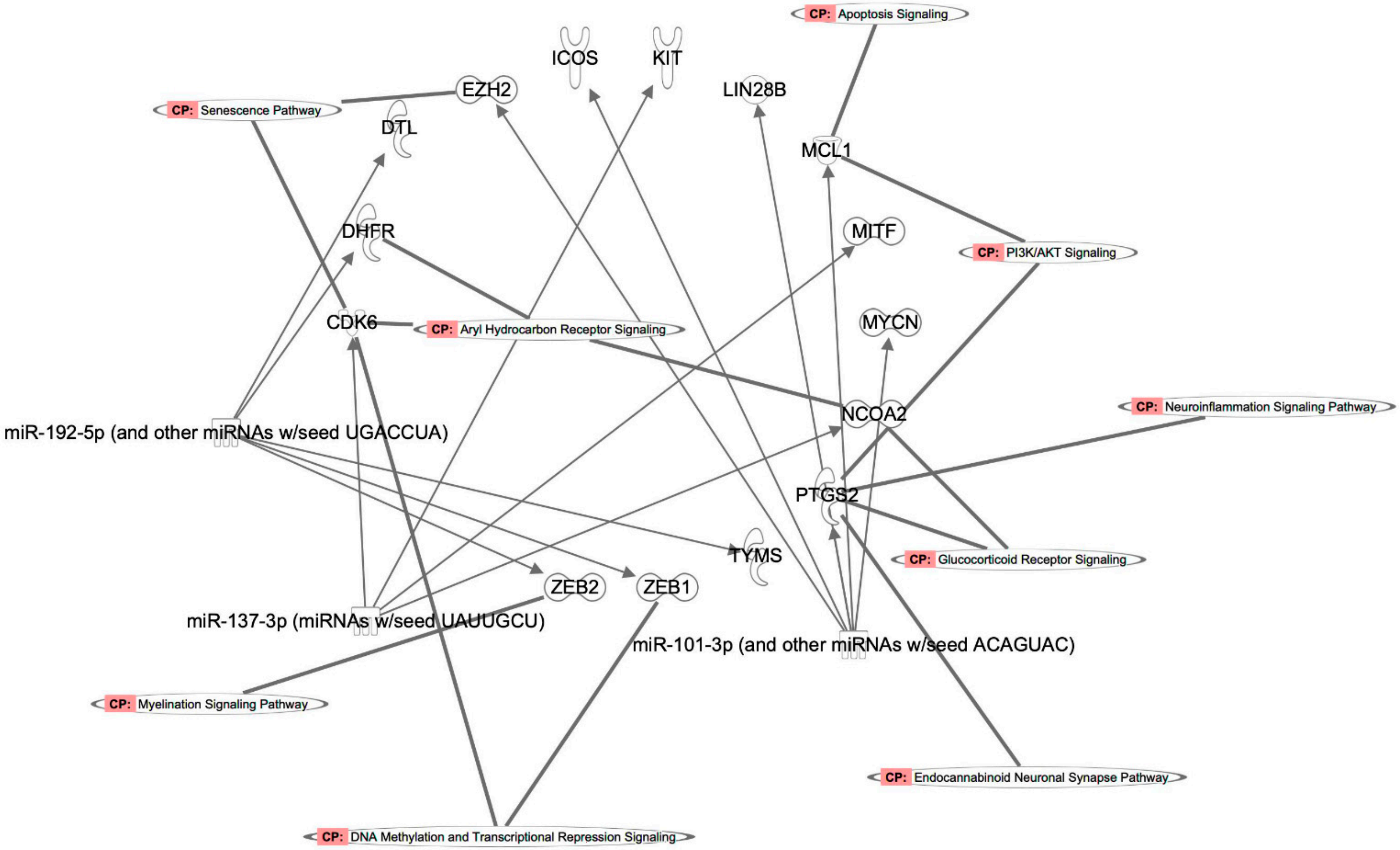

Figure S2. Cocaine withdrawal/abstinence-associated miRNA-mRNA-pathway network constructed by Ingenuity Pathway Analysis (IPA). The miRNA Target Filter function in IPA was applied to predict target genes (or mRNAs) of differentially expressed miRNAs. miRNAs were filtered to include those predicted to be target genes (or mRNAs) participating in canonical pathways associated with nervous system processes or signaling pathways. miRNAs and their target mRNAs (after filtering) along with relevant canonical pathways enriched in miRNA target mRNA sets (as determined by the IPA database) were visualized. Three such miRNAs (rno-miR101a-3p, rno-miR192-3p, and rno-miR137-3p) were identified. Pathways predicted to be regulated by these three miRNAs include: *Endocannabinioid Neuronal Synapse Pathway*, *Neuroinflammation signaling Pathway*, *PI3/Akt Signaling*, and *Apoptosis Signaling* for rno-miR101-3p; *DNA Methylation and Transcriptional Repression Signaling Pathway* and *Myelination Signaling Pathway* for rno-miR192-3p; and *Aryl Hydrocarbon Receptor Signaling and Senescence Pathway* for miR137-3p.

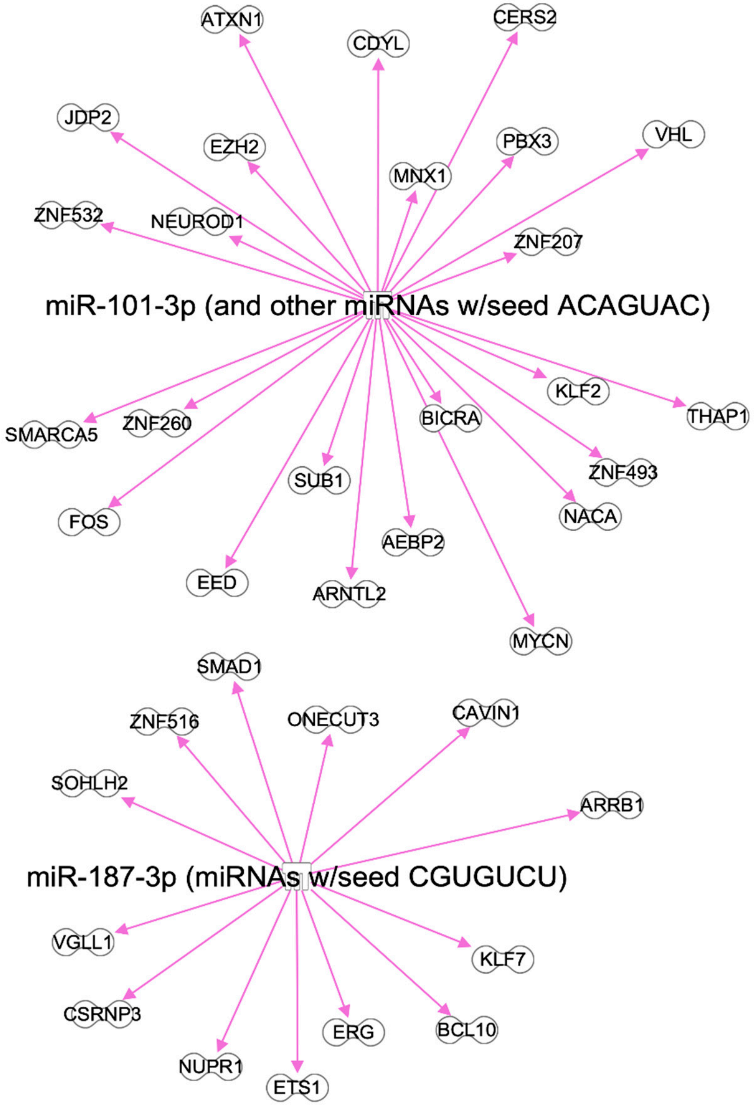

Figure S3. IPA miRNA Target Filter prediction of transcription factors (TFs) & nuclear receptors (NRs) potentially targeted by differentially expressed miRNAs in the IL-PFC of rats with protracted abstinence. miR-101a-3p and miR-187-3p are uniquely differentially expressed only during the protracted abstinence (**figure 7**). Target TFs & NRs of miR-101a-3p include FOS, NEUROD1, KLF2, and ZNFs. Target TFs & NF2 of miR-187-3p include KLF7, ZNF516, and BCL10. While mi-R101a-3p is upregulated, mi-R187-3p is downregulated (**Table 2**). It is expected that the expression of TFs & NFs targeted by mi-R101a-3p is decreased and the expression of TFs & NFs targeted by mi-R187-3p is increased.

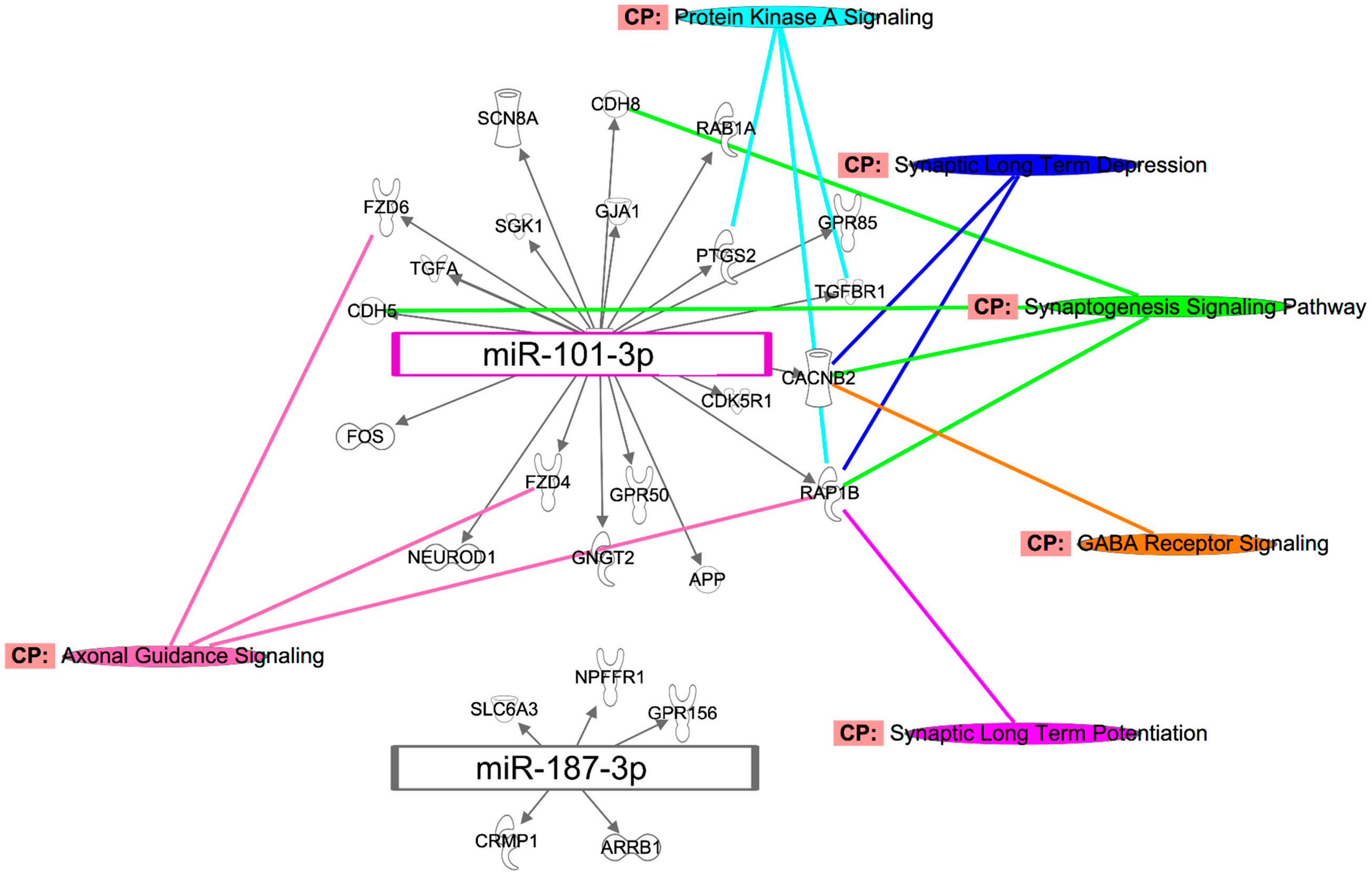

Figure S4. IPA miRNA Target Filter prediction of miRNA-mRNA-pathway regulatory networks containing miRNAs differentially expressed in the IL-PFC of rats with protracted abstinence (see **figure 7**). Putative targets of miR-101a-3p include those that are relevant to synaptogenesis signaling, synaptic long-term potentiation (LTP) and long-term depression (LTD), axonal guidance signaling, protein kinase A signaling, and GABA receptor signaling. Examples of miR-101a-3p targeted mRNAs include the calcium voltage-gated channel auxiliary subunit beta 2 gene (*CACNB2*), which is involved in synaptic LTD and GABA receptor signaling. miR-101a-3p is upregulated and hence, its targets are likely downregulated. The solute carrier family 6A member 3 gene (*SLC6A3*), which encodes the dopamine transporter, is a putative target of miR187-3p. miR187-3p is downregulated, hence the dopamine transporter gene is likely upregulated.

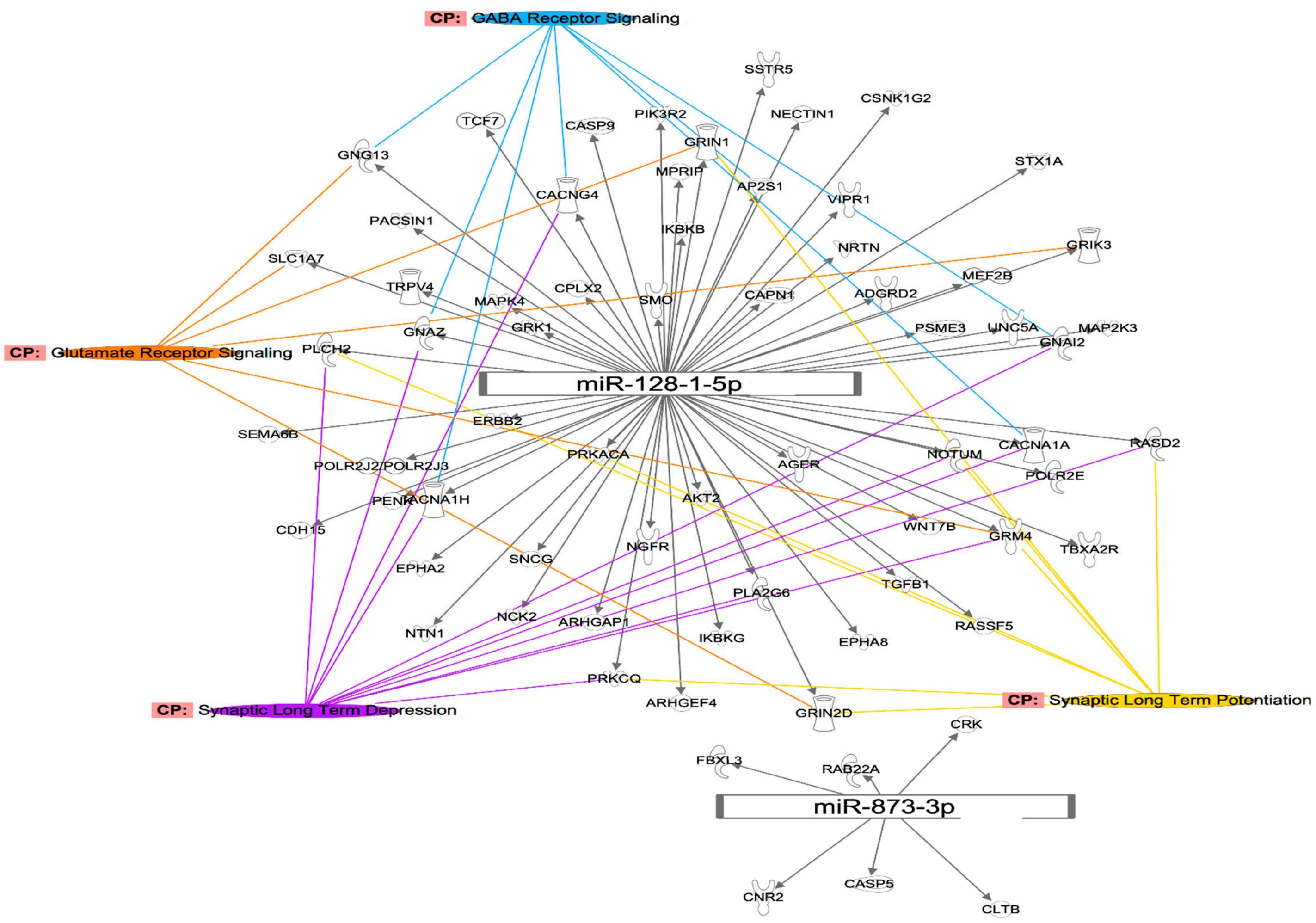

Figure S5. IPA miRNA Target Filter prediction of miRNA-mRNA-pathway regulatory networks containing miRNAs differentially expressed in the PL-PFC of rats with either withdrawal or protracted abstinence. miR-128-1-5p and miR-873-3p were differentially expressed in the PL-PFC due to either an 18-hr withdrawal or a 4-week abstinence (see **figure 7**). Examples of the predicted targets of miR-128-1-5p include the PI3 kinase receptor 2 gene (*PI3KR2*) and the phospholipase A2 group VI gene (*PLA2G6*). Predicted targets of miR-128-1-5p also include the glutamate ionotropic receptor NMDA type subunit 1 gene (*GRIN1*), the glutamate ionotropic receptor NMDA type subunit 2D gene (*GRIN2D*), the metabotropic glutamate receptor 4 gene (*GRM4*), the glutamate ionotropic receptor kainate type subunit 3 gene (*GRIK3*), and the solute carrier family 1 member 7 gene (*SLC1A7*; encoding the glutamate transporter EAAT5). These targets are involved in glutamate receptor signaling as well as synaptic LTD and LTP pathways. Additionally, miR-128-1-5p was predicted to target those genes encoding several subunits of the voltage gated calcium channels (CACNA1A and CACNA1H) and the calcium channel auxiliary subunit gamma 4 (CACNG4). miR-128-1-5p is downregulated and miR-873-3p is upregulated, and hence their targets are expected to be increased and decreased, respectively.

Table S1. The concentration and RNA integrity number (RIN) of 36 rat brain RNA samples

RNA Samples	Rat ID	Alcohol treatment	Brain Region	Conc. (ng/ul)	RNA integrity number (RIN)
1	M170	4-week abstinence	Prelimbic Prefrontal Cortex	51.4	8.9
2	M170	4-week abstinence	Infralimbic Prefrontal Cortex	47.6	8.7
3	M170	4-week abstinence	Nucleus Accumbens	23.3	9.1
4	M363	4-week abstinence	Prelimbic Prefrontal Cortex	48.0	7.4
5	M363	4-week abstinence	Infralimbic Prefrontal Cortex	30.1	7.9
6	M363	4-week abstinence	Nucleus Accumbens	38.4	8.1
7	M472	4-week abstinence	Prelimbic Prefrontal Cortex	37.4	8.5
8	M472	4-week abstinence	Infralimbic Prefrontal Cortex	42.7	9.1
9	M472	4-week abstinence	Nucleus Accumbens	41.6	9.2
10	M456	4-week abstinence	Prelimbic Prefrontal Cortex	46.8	9.1
11	M456	4-week abstinence	Infralimbic Prefrontal Cortex	21.6	8.7
12	M456	4-week abstinence	Nucleus Accumbens	50.7	8.2
13	M153	18-hr withdrawal	Prelimbic Prefrontal Cortex	33.5	9.0
14	M153	18-hr withdrawal	Infralimbic Prefrontal Cortex	55.0	9.0
15	M153	18-hr withdrawal	Nucleus Accumbens	30.9	9.0
16	M366	18-hr withdrawal	Prelimbic Prefrontal Cortex	50.0	8.8
17	M366	18-hr withdrawal	Infralimbic Prefrontal Cortex	49.6	8.2
18	M366	18-hr withdrawal	Nucleus Accumbens	52.6	8.9
19	M172	18-hr withdrawal	Prelimbic Prefrontal Cortex	48.5	9.3
20	M172	18-hr withdrawal	Infralimbic Prefrontal Cortex	60.7	8.9
21	M172	18-hr withdrawal	Nucleus Accumbens	47.5	9.1
22	M154	18-hr withdrawal	Prelimbic Prefrontal Cortex	42.2	8.8
23	M154	18-hr withdrawal	Infralimbic Prefrontal Cortex	45.8	8.9
24	M154	18-hr withdrawal	Nucleus Accumbens	53.3	9.2
25	M157	Naïve	Prelimbic Prefrontal Cortex	38.0	9.0
26	M157	Naïve	Infralimbic Prefrontal Cortex	45.7	9.2
27	M157	Naïve	Nucleus Accumbens	51.4	9.2
28	M378	Naïve	Prelimbic Prefrontal Cortex	41.8	8.5
29	M378	Naïve	Infralimbic Prefrontal Cortex	49.1	8.9
30	M378	Naïve	Nucleus Accumbens	53.0	9.1
31	M476	Naïve	Prelimbic Prefrontal Cortex	43.3	9.1
32	M476	Naïve	Infralimbic Prefrontal Cortex	42.9	9.2
33	M476	Naïve	Nucleus Accumbens	41.0	8.1
34	M480	Naïve	Prelimbic Prefrontal Cortex	44.9	8.6
35	M480	Naïve	Infralimbic Prefrontal Cortex	63.5	9.0

36	M480	Naïve	Nucleus Accumbens	39.7	9.1

Table S2. Addiction behavior Z scores and addiction index of rats with an 18-hr withdrawal or a 4-week abstinence

	Group A (4-week abstinence)				Group B (18-hr withdrawal)			
	Rat 1	Rat 2	Rat 3	Rat 4	Rat 1	Rat 2	Rat 3	Rat 4
Z ESC	1.133	0.367	-0.2	0.69	0.675	0.382	0.934	0.874
Z PR	0.123	1.325	0.445	-1.015	-0.135	0.445	-0.521	-0.135
Z shock	1.116	-0.556	0.087	2.016	-0.299	1.759	-0.17	0.988
Addiction Index	0.791	0.379	0.111	0.564	0.081	0.862	0.081	0.576

Mean±SD of addiction index in Group A: 0.461±0.288.
Mean±SD of addiction index in Group B: 0.400±0.386.
t-test of addiction index differences between Group A and Group B rats: $t = 0.25$, $P = 0.807$.

Table S3a. Correlation of addiction index with expression levels of top differentially expressed miRNAs ($|FC| \geq 2.0$ & $P < 0.05$) in three rat brain regions by partial correlation analysis

Rats	Addiction Index	Brain Region	rno-miR-101a-3p	rno-miR-1188-3p	rno-miR-136-3p	rno-miR-137-3p	rno-miR-187-3p	rno-miR-192-5p	rno-miR-292-5p	rno-miR-381-3p	rno-miR-382-3p	rno-miR-448-3p	rno-miR-499-5p	rno-miR-551b-3p	rno-miR-582-3p	rno-miR-666-3p	rno-miR-764-5p	rno-miR-872-5p
A_Rat 1	0.791	IL-PFC	-0.032	0.512	0.201	-0.643	-0.970	-1.086	-0.460	0.705	-0.246	-0.834	-0.262	-0.983	-0.841	-1.349	0.781	-0.487
A_Rat 2	0.379	IL-PFC	0.432	-0.772	0.577	-0.203	1.417	0.601	0.034	1.503	1.173	-1.074	0.361	-0.249	0.368	-0.219	-0.913	0.314
A_Rat 3	0.111	IL-PFC	-0.357	-0.072	-0.306	0.250	0.038	-0.545	-0.346	0.112	-0.660	0.309	0.832	0.551	1.044	0.673	-0.837	0.414
A_Rat 4	0.564	IL-PFC	-0.001	0.450	-0.421	0.452	-0.512	1.076	0.645	-2.253	0.068	1.584	-1.029	0.541	-0.693	0.886	1.107	-0.301
B_Rat 1	0.081	IL-PFC	0.157	0.975	-0.675	-0.842	3.567	-0.205	1.695	-2.181	-1.589	2.368	-1.697	-0.276	-3.313	-0.420	3.015	-0.844
B_Rat 2	0.862	IL-PFC	0.103	-0.756	0.680	1.252	-0.107	-0.681	1.466	-0.435	2.263	-0.828	1.578	-2.191	1.392	1.256	-0.597	0.909
B_Rat 3	0.081	IL-PFC	-0.735	0.266	-0.787	-1.393	-1.939	0.943	-1.943	1.023	0.139	-2.081	0.744	0.609	0.423	0.364	-2.330	-0.406
B_Rat 4	0.576	IL-PFC	0.366	-0.423	0.555	1.224	-1.498	0.037	-1.284	1.526	-0.782	0.580	-0.473	1.906	1.656	-1.251	0.101	0.468
		Partial correlation of addiction index with miRNA expression levels in the infralimbic prefrontal cortex (IL-PFC):																
		estimate	0.444	-0.367	0.732	0.601	-0.341	-0.371	0.202	0.082	0.539	-0.154	0.184	-0.453	0.312	-0.090	0.074	0.407
		statistic	1.110	-0.882	2.400	1.680	-0.812	-0.894	0.461	0.184	1.431	-0.349	0.418	-1.135	0.734	-0.203	0.165	0.996
		p-value	0.318	0.418	0.062	0.154	0.454	0.412	0.664	0.862	0.212	0.742	0.693	0.308	0.496	0.847	0.875	0.365

Rats	Addiction Index	Brain Region	rno-miR-101a-3p	rno-miR-1188-3p	rno-miR-136-3p	rno-miR-137-3p	rno-miR-187-3p	rno-miR-192-5p	rno-miR-292-5p	rno-miR-381-3p	rno-miR-382-3p	rno-miR-448-3p	rno-miR-499-5p	rno-miR-551b-3p	rno-miR-582-3p	rno-miR-666-3p	rno-miR-764-5p	rno-miR-872-5p
A_Rat 1	0.791	PL-PFC	-1.066	0.439	-1.039	-0.196	0.032	-1.339	0.394	0.888	0.302	0.283	1.036	0.202	0.040	0.452	-1.023	0.244
A_Rat 2	0.379	PL-PFC	0.984	-0.013	0.067	0.067	-0.069	0.636	-0.588	0.374	0.272	1.177	1.250	0.045	0.237	-0.700	-0.519	0.556
A_Rat 3	0.111	PL-PFC	-1.151	-0.702	-0.388	1.136	0.110	0.203	-0.617	-2.007	0.408	0.880	-0.208	-0.059	0.566	-0.962	0.695	-0.097
A_Rat 4	0.564	PL-PFC	1.260	0.392	1.254	-0.926	-0.068	0.355	1.443	0.871	-0.855	-2.361	-1.734	-0.125	-0.798	1.332	0.825	-0.606
B_Rat 1	0.081	PL-PFC	-0.503	1.665	-0.165	-0.359	-0.027	-0.827	1.027	0.172	-1.273	0.159	-0.590	0.169	-0.379	1.359	0.946	-0.788
B_Rat 2	0.862	PL-PFC	1.179	1.802	0.527	1.174	0.052	0.298	1.178	-0.144	1.563	-0.028	0.825	1.234	1.229	-2.561	-0.454	0.608
B_Rat 3	0.081	PL-PFC	0.297	-4.374	-0.028	-1.739	0.127	-0.076	-1.230	0.190	-1.958	-0.698	-0.901	-1.719	-0.940	1.505	-1.258	-0.679
B_Rat 4	0.576	PL-PFC	-0.915	0.877	-0.336	0.628	-0.142	0.644	-1.173	-0.222	1.343	0.453	0.462	-0.041	-0.165	-0.385	0.589	0.759
		Partial correlation of addiction index with miRNA expression levels in the prelimbic prefrontal cortex (PL-PFC):																
		estimate	0.239	0.529	0.109	0.341	-0.298	0.007	0.396	0.413	0.671	-0.130	0.462	0.634	0.427	-0.472	-0.235	0.640
		statistic	0.551	1.392	0.245	0.812	-0.697	0.015	0.964	1.014	2.024	-0.292	1.166	1.835	1.056	-1.196	-0.540	1.861
		p-value	0.605	0.223	0.816	0.454	0.517	0.989	0.379	0.357	0.099	0.782	0.296	0.126	0.339	0.285	0.612	0.122

Rats	Addiction Index	Brain Region	rno-miR-101a-3p	rno-miR-1188-3p	rno-miR-136-3p	rno-miR-137-3p	rno-miR-187-3p	rno-miR-192-5p	rno-miR-292-5p	rno-miR-381-3p	rno-miR-382-3p	rno-miR-448-3p	rno-miR-499-5p	rno-miR-551b-3p	rno-miR-582-3p	rno-miR-666-3p	rno-miR-764-5p	rno-miR-872-5p
A_Rat 1	0.791	NAc	-0.667	0.630	-0.377	-0.412	0.924	-0.870	0.534	-0.417	0.067	-0.246	0.121	0.100	0.140	0.076	-0.376	-0.319
A_Rat 2	0.379	NAc	-2.222	0.577	-1.308	-2.016	-0.381	-1.787	-0.032	-1.168	-1.892	-0.910	-2.265	-1.073	-1.660	1.199	0.564	-2.278
A_Rat 3	0.111	NAc	1.027	0.087	0.885	0.435	-0.667	0.401	-1.266	0.647	0.615	0.315	1.296	0.119	0.278	-0.585	-0.299	0.663
A_Rat 4	0.564	NAc	1.833	-1.245	0.704	1.957	0.135	2.251	0.588	0.832	1.005	0.808	0.540	0.887	1.237	-0.618	0.075	1.940
B_Rat 1	0.081	NAc	0.908	0.424	0.292	-0.105	0.514	-0.067	1.573	0.392	-0.673	0.440	0.267	1.467	-0.956	0.519	0.160	-0.113

B_Rat 2	0.862	NAc	-1.595	-0.175	-1.284	-1.174	0.405	-1.204	1.699	-0.768	-0.633	-0.724	-1.133	-1.216	0.062	0.577	0.106	-1.021
B_Rat 3	0.081	NAc	0.856	-0.212	0.979	0.549	-0.167	1.595	-0.863	0.845	0.136	-0.078	0.894	0.086	0.168	-1.169	-0.153	0.644
B_Rat 4	0.576	NAc	-0.159	-0.062	0.209	0.808	-0.756	-0.298	-2.444	-0.399	1.224	0.398	0.093	-0.169	0.786	0.001	-0.132	0.529
		Partial correlation of addiction index with miRNA expression levels in the nucleus accumbens (NAc):																
		estimate	-0.493	-0.119	-0.602	-0.170	0.400	-0.376	0.257	-0.581	0.071	-0.312	-0.413	-0.463	0.310	0.319	-0.052	-0.178
		statistic	-1.268	-0.267	-1.686	-0.387	0.976	-0.909	0.594	-1.598	0.159	-0.734	-1.015	-1.168	0.730	0.753	-0.116	-0.405
		p-value	0.261	0.800	0.153	0.715	0.374	0.405	0.578	0.171	0.880	0.496	0.357	0.296	0.498	0.485	0.912	0.702

IL-PFC: Infralimbic prefrontal cortex; PL-PFC: Prelimbic prefrontal cortex; NAc: Nucleus accumbens.
Normalized expression levels of miRNAs are listed under the name of each miRNA.

Table S3b. Correlation of Z ESC with expression levels of top differentially expressed miRNAs (|FC| ≥ 2.0 & *P* < 0.05) in three rat brain regions by partial correlation analysis

Rats	Addiction Index	Brain Region	rno-miR-101a-3p	rno-miR-1188-3p	rno-miR-136-3p	rno-miR-137-3p	rno-miR-187-3p	rno-miR-192-5p	rno-miR-292-5p	rno-miR-381-3p	rno-miR-382-3p	rno-miR-448-3p	rno-miR-499-5p	rno-miR-551b-3p	rno-miR-582-3p	rno-miR-666-3p	rno-miR-764-5p	rno-miR-872-5p
A_Rat 1	1.133	IL-PFC	-0.032	0.512	0.201	-0.643	-0.970	-1.086	-0.460	0.705	-0.246	-0.834	-0.262	-0.983	-0.841	-1.349	0.781	-0.487
A_Rat 2	0.367	IL-PFC	0.432	-0.772	0.577	-0.203	1.417	0.601	0.034	1.503	1.173	-1.074	0.361	-0.249	0.368	-0.219	-0.913	0.314
A_Rat 3	-0.200	IL-PFC	-0.357	-0.072	-0.306	0.250	0.038	-0.545	-0.346	0.112	-0.660	0.309	0.832	0.551	1.044	0.673	-0.837	0.414
A_Rat 4	0.690	IL-PFC	-0.001	0.450	-0.421	0.452	-0.512	1.076	0.645	-2.253	0.068	1.584	-1.029	0.541	-0.693	0.886	1.107	-0.301
B_Rat 1	0.675	IL-PFC	0.157	0.975	-0.675	-0.842	3.567	-0.205	1.695	-2.181	-1.589	2.368	-1.697	-0.276	-3.313	-0.420	3.015	-0.844
B_Rat 2	0.382	IL-PFC	0.103	-0.756	0.680	1.252	-0.107	-0.681	1.466	-0.435	2.263	-0.828	1.578	-2.191	1.392	1.256	-0.597	0.909
B_Rat 3	0.934	IL-PFC	-0.735	0.266	-0.787	-1.393	-1.939	0.943	-1.943	1.023	0.139	-2.081	0.744	0.609	0.423	0.364	-2.330	-0.406
B_Rat 4	0.874	IL-PFC	0.366	-0.423	0.555	1.224	-1.498	0.037	-1.284	1.526	-0.782	0.580	-0.473	1.906	1.656	-1.251	0.101	0.468
		Partial correlation of Z ESC with miRNA expression levels in the infralimbic prefrontal cortex (IL-PFC):																
		estimate	0.027	-0.116	-0.059	-0.622	-0.271	0.120	0.048	0.829	0.027	-0.116	-0.059	-0.622	-0.271	0.120	0.048	0.829
		statistic	0.060	-0.261	-0.132	-1.776	-0.629	0.269	0.108	3.310	0.060	-0.261	-0.132	-1.776	-0.629	0.269	0.108	3.310
		p-value	0.954	0.804	0.900	0.136	0.557	0.798	0.918	**0.021**	0.542	0.427	0.961	0.526	0.165	0.150	0.386	0.842

Rats	Addiction Index	Brain Region	rno-miR-101a-3p	rno-miR-1188-3p	rno-miR-136-3p	rno-miR-137-3p	rno-miR-187-3p	rno-miR-192-5p	rno-miR-292-5p	rno-miR-381-3p	rno-miR-382-3p	rno-miR-448-3p	rno-miR-499-5p	rno-miR-551b-3p	rno-miR-582-3p	rno-miR-666-3p	rno-miR-764-5p	rno-miR-872-5p
A_Rat 1	1.133	PL-PFC	-1.066	0.439	-1.039	-0.196	0.032	-1.339	0.394	0.888	0.302	0.283	1.036	0.202	0.040	0.452	-1.023	0.244
A_Rat 2	0.367	PL-PFC	0.984	-0.013	0.067	0.067	-0.069	0.636	-0.588	0.374	0.272	1.177	1.250	0.045	0.237	-0.700	-0.519	0.556
A_Rat 3	-0.200	PL-PFC	-1.151	-0.702	-0.388	1.136	0.110	0.203	-0.617	-2.007	0.408	0.880	-0.208	-0.059	0.566	-0.962	0.695	-0.097
A_Rat 4	0.690	PL-PFC	1.260	0.392	1.254	-0.926	-0.068	0.355	1.443	0.871	-0.855	-2.361	-1.734	-0.125	-0.798	1.332	0.825	-0.606
B_Rat 1	0.675	PL-PFC	-0.503	1.665	-0.165	-0.359	-0.027	-0.827	1.027	0.172	-1.273	0.159	-0.590	0.169	-0.379	1.359	0.946	-0.788
B_Rat 2	0.382	PL-PFC	1.179	1.802	0.527	1.174	0.052	0.298	1.178	-0.144	1.563	-0.028	0.825	1.234	1.229	-2.561	-0.454	0.608
B_Rat 3	0.934	PL-PFC	0.297	-4.374	-0.028	-1.739	0.127	-0.076	-1.230	0.190	-1.958	-0.698	-0.901	-1.719	-0.940	1.505	-1.258	-0.679
B_Rat 4	0.874	PL-PFC	-0.915	0.877	-0.336	0.628	-0.142	0.644	-1.173	-0.222	1.343	0.453	0.462	-0.041	-0.165	-0.385	0.589	0.759
		Partial correlation of Z ESC with miRNA expression levels in the prelimbic prefrontal cortex (PL-PFC):																
		estimate	-0.073	0.429	-0.202	-0.358	-0.340	-0.493	-0.314	0.102	-0.183	-0.115	-0.451	0.089	-0.313	0.592	0.220	-0.582
		statistic	-0.164	1.061	-0.460	-0.857	-0.810	-1.266	-0.739	0.230	-0.417	-0.260	-1.130	0.199	-0.736	1.644	0.504	-1.600
		p-value	0.876	0.337	0.665	0.431	0.455	0.261	0.493	0.827	0.694	0.806	0.310	0.850	0.495	0.161	0.636	0.170

Rats	Addiction Index	Brain Region	rno-miR-101a-3p	rno-miR-1188-3p	rno-miR-136-3p	rno-miR-137-3p	rno-miR-187-3p	rno-miR-192-5p	rno-miR-292-5p	rno-miR-381-3p	rno-miR-382-3p	rno-miR-448-3p	rno-miR-499-5p	rno-miR-551b-3p	rno-miR-582-3p	rno-miR-666-3p	rno-miR-764-5p	rno-miR-872-5p
A_Rat 1	1.133	NAc	-0.667	0.630	-0.377	-0.412	0.924	-0.870	0.534	-0.417	0.067	-0.246	0.121	0.100	0.140	0.076	-0.376	-0.319
A_Rat 2	0.367	NAc	-2.222	0.577	-1.308	-2.016	-0.381	-1.787	-0.032	-1.168	-1.892	-0.910	-2.265	-1.073	-1.660	1.199	0.564	-2.278
A_Rat 3	-0.200	NAc	1.027	0.087	0.885	0.435	-0.667	0.401	-1.266	0.647	0.615	0.315	1.296	0.119	0.278	-0.585	-0.299	0.663
A_Rat 4	0.690	NAc	1.833	-1.245	0.704	1.957	0.135	2.251	0.588	0.832	1.005	0.808	0.540	0.887	1.237	-0.618	0.075	1.940

B_Rat 1	0.675	NAc	0.908	0.424	0.292	-0.105	0.514	-0.067	1.573	0.392	-0.673	0.440	0.267	1.467	-0.956	0.519	0.160	-0.113
B_Rat 2	0.382	NAc	-1.595	-0.175	-1.284	-1.174	0.405	-1.204	1.699	-0.768	-0.633	-0.724	-1.133	-1.216	0.062	0.577	0.106	-1.021
B_Rat 3	0.934	NAc	0.856	-0.212	0.979	0.549	-0.167	1.595	-0.863	0.845	0.136	-0.078	0.894	0.086	0.168	-1.169	-0.153	0.644
B_Rat 4	0.874	NAc	-0.159	-0.062	0.209	0.808	-0.756	-0.298	-2.444	-0.399	1.224	0.398	0.093	-0.169	0.786	0.001	-0.132	0.529
		Partial correlation of Z ESC with miRNA expression levels in the nucleus accumbens (NAc):																
		estimate	0.040	0.005	0.044	0.208	0.486	0.140	0.033	-0.019	0.179	0.100	0.046	0.250	0.204	-0.133	-0.220	0.162
		statistic	0.090	0.012	0.099	0.475	1.245	0.315	0.074	-0.042	0.408	0.225	0.103	0.577	0.467	-0.301	-0.504	0.366
		p-value	0.932	0.991	0.925	0.655	0.268	0.765	0.944	0.968	0.700	0.831	0.922	0.589	0.660	0.776	0.635	0.729

IL-PFC: Infralimbic prefrontal cortex; PL-PFC: Prelimbic prefrontal cortex; NAc: Nucleus accumbens.
Normalized expression levels of miRNAs are listed under the name of each miRNA.

Table S3c. Correlation of Z PR with expression levels of top differentially expressed miRNAs (|FC| ≥ 2.0 & $P < 0.05$) in three rat brain regions by partial correlation analysis

Rats	Addiction Index	Brain Region	rno-miR-101a-3p	rno-miR-1188-3p	rno-miR-136-3p	rno-miR-137-3p	rno-miR-187-3p	rno-miR-192-5p	rno-miR-292-5p	rno-miR-381-3p	rno-miR-382-3p	rno-miR-448-3p	rno-miR-499-5p	rno-miR-551b-3p	rno-miR-582-3p	rno-miR-666-3p	rno-miR-764-5p	rno-miR-872-5p
A_Rat 1	0.123	IL-PFC	-0.032	0.512	0.201	-0.643	-0.970	-1.086	-0.460	0.705	-0.246	-0.834	-0.262	-0.983	-0.841	-1.349	0.781	-0.487
A_Rat 2	1.325	IL-PFC	0.432	-0.772	0.577	-0.203	1.417	0.601	0.034	1.503	1.173	-1.074	0.361	-0.249	0.368	-0.219	-0.913	0.314
A_Rat 3	0.445	IL-PFC	-0.357	-0.072	-0.306	0.250	0.038	-0.545	-0.346	0.112	-0.660	0.309	0.832	0.551	1.044	0.673	-0.837	0.414
A_Rat 4	-1.015	IL-PFC	-0.001	0.450	-0.421	0.452	-0.512	1.076	0.645	-2.253	0.068	1.584	-1.029	0.541	-0.693	0.886	1.107	-0.301
B_Rat 1	-0.135	IL-PFC	0.157	0.975	-0.675	-0.842	3.567	-0.205	1.695	-2.181	-1.589	2.368	-1.697	-0.276	-3.313	-0.420	3.015	-0.844
B_Rat 2	0.445	IL-PFC	0.103	-0.756	0.680	1.252	-0.107	-0.681	1.466	-0.435	2.263	-0.828	1.578	-2.191	1.392	1.256	-0.597	0.909
B_Rat 3	-0.521	IL-PFC	-0.735	0.266	-0.787	-1.393	-1.939	0.943	-1.943	1.023	0.139	-2.081	0.744	0.609	0.423	0.364	-2.330	-0.406
B_Rat 4	-0.135	IL-PFC	0.366	-0.423	0.555	1.224	-1.498	0.037	-1.284	1.526	-0.782	0.580	-0.473	1.906	1.656	-1.251	0.101	0.468
		Partial correlation of Z ESC with miRNA expression levels in the infralimbic prefrontal cortex (IL-PFC):																
		estimate	0.410	0.217	0.624	0.592	0.022	-0.381	-0.278	-0.310	0.522	0.878	0.838	0.406	0.719	-0.100	-0.260	0.643
		statistic	1.004	0.498	1.786	1.644	0.048	-0.921	-0.647	-0.728	1.367	4.104	3.431	0.994	2.313	-0.225	-0.602	1.876
		p-value	0.361	0.640	0.134	0.161	0.963	0.399	0.546	0.499	0.230	**0.009**	0.019	0.366	0.069	0.831	0.573	0.119

Rats	Addiction Index	Brain Region	rno-miR-101a-3p	rno-miR-1188-3p	rno-miR-136-3p	rno-miR-137-3p	rno-miR-187-3p	rno-miR-192-5p	rno-miR-292-5p	rno-miR-381-3p	rno-miR-382-3p	rno-miR-448-3p	rno-miR-499-5p	rno-miR-551b-3p	rno-miR-582-3p	rno-miR-666-3p	rno-miR-764-5p	rno-miR-872-5p
A_Rat 1	0.123	PL-PFC	-1.066	0.439	-1.039	-0.196	0.032	-1.339	0.394	0.888	0.302	0.283	1.036	0.202	0.040	0.452	-1.023	0.244
A_Rat 2	1.325	PL-PFC	0.984	-0.013	0.067	0.067	-0.069	0.636	-0.588	0.374	0.272	1.177	1.250	0.045	0.237	-0.700	-0.519	0.556
A_Rat 3	0.445	PL-PFC	-1.151	-0.702	-0.388	1.136	0.110	0.203	-0.617	-2.007	0.408	0.880	-0.208	-0.059	0.566	-0.962	0.695	-0.097
A_Rat 4	-1.015	PL-PFC	1.260	0.392	1.254	-0.926	-0.068	0.355	1.443	0.871	-0.855	-2.361	-1.734	-0.125	-0.798	1.332	0.825	-0.606
B_Rat 1	-0.135	PL-PFC	-0.503	1.665	-0.165	-0.359	-0.027	-0.827	1.027	0.172	-1.273	0.159	-0.590	0.169	-0.379	1.359	0.946	-0.788
B_Rat 2	0.445	PL-PFC	1.179	1.802	0.527	1.174	0.052	0.298	1.178	-0.144	1.563	-0.028	0.825	1.234	1.229	-2.561	-0.454	0.608
B_Rat 3	-0.521	PL-PFC	0.297	-4.374	-0.028	-1.739	0.127	-0.076	-1.230	0.190	-1.958	-0.698	-0.901	-1.719	-0.940	1.505	-1.258	-0.679
B_Rat 4	-0.135	PL-PFC	-0.915	0.877	-0.336	0.628	-0.142	0.644	-1.173	-0.222	1.343	0.453	0.462	-0.041	-0.165	-0.385	0.589	0.759
		Partial correlation of Z ESC with miRNA expression levels in the prelimbic prefrontal cortex (PL-PFC):																
		estimate	-0.012	-0.662	-0.352	0.128	0.352	0.187	0.134	0.516	0.407	-0.375	0.486	-0.380	0.286	-0.694	-0.271	0.535
		statistic	-0.026	-1.975	-0.841	0.289	0.841	0.427	0.302	1.346	0.995	-0.906	1.242	-0.919	0.668	-2.157	-0.629	1.415
		p-value	0.980	0.105	0.439	0.784	0.439	0.687	0.775	0.236	0.365	0.407	0.269	0.400	0.534	0.083	0.557	0.216

Rats	Addiction Index	Brain Region	rno-miR-101a-3p	rno-miR-1188-3p	rno-miR-136-3p	rno-miR-137-3p	rno-miR-187-3p	rno-miR-192-5p	rno-miR-292-5p	rno-miR-381-3p	rno-miR-382-3p	rno-miR-448-3p	rno-miR-499-5p	rno-miR-551b-3p	rno-miR-582-3p	rno-miR-666-3p	rno-miR-764-5p	rno-miR-872-5p
A_Rat 1	0.123	NAc	-0.667	0.630	-0.377	-0.412	0.924	-0.870	0.534	-0.417	0.067	-0.246	0.121	0.100	0.140	0.076	-0.376	-0.319
A_Rat 2	1.325	NAc	-2.222	0.577	-1.308	-2.016	-0.381	-1.787	-0.032	-1.168	-1.892	-0.910	-2.265	-1.073	-1.660	1.199	0.564	-2.278
A_Rat 3	0.445	NAc	1.027	0.087	0.885	0.435	-0.667	0.401	-1.266	0.647	0.615	0.315	1.296	0.119	0.278	-0.585	-0.299	0.663
A_Rat 4	-1.015	NAc	1.833	-1.245	0.704	1.957	0.135	2.251	0.588	0.832	1.005	0.808	0.540	0.887	1.237	-0.618	0.075	1.940
B_Rat 1	-0.135	NAc	0.908	0.424	0.292	-0.105	0.514	-0.067	1.573	0.392	-0.673	0.440	0.267	1.467	-0.956	0.519	0.160	-0.113

B_Rat 2	0.445	NAc	-1.595	-0.175	-1.284	-1.174	0.405	-1.204	1.699	-0.768	-0.633	-0.724	-1.133	-1.216	0.062	0.577	0.106	-1.021
B_Rat 3	-0.521	NAc	0.856	-0.212	0.979	0.549	-0.167	1.595	-0.863	0.845	0.136	-0.078	0.894	0.086	0.168	-1.169	-0.153	0.644
B_Rat 4	-0.135	NAc	-0.159	-0.062	0.209	0.808	-0.756	-0.298	-2.444	-0.399	1.224	0.398	0.093	-0.169	0.786	0.001	-0.132	0.529
		Partial correlation of Z ESC with miRNA expression levels in the nucleus accumbens (NAc):																
		estimate	-0.840	0.732	-0.733	-0.916	-0.185	-0.893	0.033	-0.777	-0.738	-0.799	-0.707	-0.701	-0.757	0.744	0.431	-0.918
		statistic	-3.463	2.401	-2.407	-5.115	-0.420	-4.449	0.074	-2.764	-2.448	-2.975	-2.238	-2.198	-2.592	2.487	1.069	-5.168
		p-value	**0.018**	0.062	0.061	**0.004**	0.692	**0.007**	0.944	**0.040**	0.058	**0.031**	0.075	0.079	**0.049**	0.055	0.334	**0.004**

IL-PFC: Infralimbic prefrontal cortex; PL-PFC: Prelimbic prefrontal cortex; NAc: Nucleus accumbens.
Normalized expression levels of miRNAs are listed under the name of each miRNA.

Table S3d. Correlation of Z Shock with expression levels of top differentially expressed miRNAs (|FC| ≥ 2.0 & $P < 0.05$) in three rat brain regions by partial correlation analysis

Rats	Addiction Index	Brain Region	rno-miR-101a-3p	rno-miR-1188-3p	rno-miR-136-3p	rno-miR-137-3p	rno-miR-187-3p	rno-miR-192-5p	rno-miR-292-5p	rno-miR-381-3p	rno-miR-382-3p	rno-miR-448-3p	rno-miR-499-5p	rno-miR-551b-3p	rno-miR-582-3p	rno-miR-666-3p	rno-miR-764-5p	rno-miR-872-5p
A_Rat 1	1.116	IL-PFC	-0.032	0.512	0.201	-0.643	-0.970	-1.086	-0.460	0.705	-0.246	-0.834	-0.262	-0.983	-0.841	-1.349	0.781	-0.487
A_Rat 2	-0.556	IL-PFC	0.432	-0.772	0.577	-0.203	1.417	0.601	0.034	1.503	1.173	-1.074	0.361	-0.249	0.368	-0.219	-0.913	0.314
A_Rat 3	0.087	IL-PFC	-0.357	-0.072	-0.306	0.250	0.038	-0.545	-0.346	0.112	-0.660	0.309	0.832	0.551	1.044	0.673	-0.837	0.414
A_Rat 4	2.016	IL-PFC	-0.001	0.450	-0.421	0.452	-0.512	1.076	0.645	-2.253	0.068	1.584	-1.029	0.541	-0.693	0.886	1.107	-0.301
B_Rat 1	-0.299	IL-PFC	0.157	0.975	-0.675	-0.842	3.567	-0.205	1.695	-2.181	-1.589	2.368	-1.697	-0.276	-3.313	-0.420	3.015	-0.844
B_Rat 2	1.759	IL-PFC	0.103	-0.756	0.680	1.252	-0.107	-0.681	1.466	-0.435	2.263	-0.828	1.578	-2.191	1.392	1.256	-0.597	0.909
B_Rat 3	-0.170	IL-PFC	-0.735	0.266	-0.787	-1.393	-1.939	0.943	-1.943	1.023	0.139	-2.081	0.744	0.609	0.423	0.364	-2.330	-0.406
B_Rat 4	0.988	IL-PFC	0.366	-0.423	0.555	1.224	-1.498	0.037	-1.284	1.526	-0.782	0.580	-0.473	1.906	1.656	-1.251	0.101	0.468
		Partial correlation of Z ESC with miRNA expression levels in the infralimbic prefrontal cortex (IL-PFC):																
		estimate	0.132	0.406	0.294	0.171	-0.191	-0.141	0.556	0.275	0.398	-0.590	-0.129	0.448	0.151	0.231	0.115	0.207
		statistic	0.297	0.992	0.689	0.387	-0.436	-0.319	1.495	0.639	0.971	-1.633	0.291	1.120	0.342	0.530	0.259	0.472
		p-value	0.778	0.367	0.522	0.714	0.681	0.763	0.195	0.551	0.376	0.163	0.783	0.314	0.746	0.619	0.806	0.656

Rats	Addiction Index	Brain Region	rno-miR-101a-3p	rno-miR-1188-3p	rno-miR-136-3p	rno-miR-137-3p	rno-miR-187-3p	rno-miR-192-5p	rno-miR-292-5p	rno-miR-381-3p	rno-miR-382-3p	rno-miR-448-3p	rno-miR-499-5p	rno-miR-551b-3p	rno-miR-582-3p	rno-miR-666-3p	rno-miR-764-5p	rno-miR-872-5p
A_Rat 1	1.116	PL-PFC	-1.066	0.439	-1.039	-0.196	0.032	-1.339	0.394	0.888	0.302	0.283	1.036	0.202	0.040	0.452	-1.023	0.244
A_Rat 2	-0.556	PL-PFC	0.984	-0.013	0.067	0.067	-0.069	0.636	-0.588	0.374	0.272	1.177	1.250	0.045	0.237	-0.700	-0.519	0.556
A_Rat 3	0.087	PL-PFC	-1.151	-0.702	-0.388	1.136	0.110	0.203	-0.617	-2.007	0.408	0.880	-0.208	-0.059	0.566	-0.962	0.695	-0.097
A_Rat 4	2.016	PL-PFC	1.260	0.392	1.254	-0.926	-0.068	0.355	1.443	0.871	-0.855	-2.361	-1.734	-0.125	-0.798	1.332	0.825	-0.606
B_Rat 1	-0.299	PL-PFC	-0.503	1.665	-0.165	-0.359	-0.027	-0.827	1.027	0.172	-1.273	0.159	-0.590	0.169	-0.379	1.359	0.946	-0.788
B_Rat 2	1.759	PL-PFC	1.179	1.802	0.527	1.174	0.052	0.298	1.178	-0.144	1.563	-0.028	0.825	1.234	1.229	-2.561	-0.454	0.608
B_Rat 3	-0.170	PL-PFC	0.297	-4.374	-0.028	-1.739	0.127	-0.076	-1.230	0.190	-1.958	-0.698	-0.901	-1.719	-0.940	1.505	-1.258	-0.679
B_Rat 4	0.988	PL-PFC	-0.915	0.877	-0.336	0.628	-0.142	0.644	-1.173	-0.222	1.343	0.453	0.462	-0.041	-0.165	-0.385	0.589	0.759
		Partial correlation of Z ESC with miRNA expression levels in the prelimbic prefrontal cortex (PL-PFC):																
		estimate	0.269	-0.068	0.433	0.636	-0.435	0.077	0.230	-0.323	0.311	0.161	0.023	-0.208	0.229	-0.213	0.170	0.258
		statistic	0.624	-0.151	1.075	1.845	-1.081	0.174	0.528	-0.762	0.732	0.364	0.052	-0.474	0.527	-0.488	0.385	0.596
		p-value	0.560	0.886	0.332	0.124	0.329	0.869	0.620	0.480	0.497	0.731	0.961	0.655	0.621	0.647	0.716	0.577

Rats	Addiction Index	Brain Region	rno-miR-101a-3p	rno-miR-1188-3p	rno-miR-136-3p	rno-miR-137-3p	rno-miR-187-3p	rno-miR-192-5p	rno-miR-292-5p	rno-miR-381-3p	rno-miR-382-3p	rno-miR-448-3p	rno-miR-499-5p	rno-miR-551b-3p	rno-miR-582-3p	rno-miR-666-3p	rno-miR-764-5p	rno-miR-872-5p
A_Rat 1	1.116	NAc	-0.667	0.630	-0.377	-0.412	0.924	-0.870	0.534	-0.417	0.067	-0.246	0.121	0.100	0.140	0.076	-0.376	-0.319
A_Rat 2	-0.556	NAc	-2.222	0.577	-1.308	-2.016	-0.381	-1.787	-0.032	-1.168	-1.892	-0.910	-2.265	-1.073	-1.660	1.199	0.564	-2.278
A_Rat 3	0.087	NAc	1.027	0.087	0.885	0.435	-0.667	0.401	-1.266	0.647	0.615	0.315	1.296	0.119	0.278	-0.585	-0.299	0.663
A_Rat 4	2.016	NAc	1.833	-1.245	0.704	1.957	0.135	2.251	0.588	0.832	1.005	0.808	0.540	0.887	1.237	-0.618	0.075	1.940
B_Rat 1	-0.299	NAc	0.908	0.424	0.292	-0.105	0.514	-0.067	1.573	0.392	-0.673	0.440	0.267	1.467	-0.956	0.519	0.160	-0.113

B_Rat 2	1.759	NAc	-1.595	-0.175	-1.284	-1.174	0.405	-1.204	1.699	-0.768	-0.633	-0.724	-1.133	-1.216	0.062	0.577	0.106	-1.021
B_Rat 3	-0.170	NAc	0.856	-0.212	0.979	0.549	-0.167	1.595	-0.863	0.845	0.136	-0.078	0.894	0.086	0.168	-1.169	-0.153	0.644
B_Rat 4	0.988	NAc	-0.159	-0.062	0.209	0.808	-0.756	-0.298	-2.444	-0.399	1.224	0.398	0.093	-0.169	0.786	0.001	-0.132	0.529
		Partial correlation of Z ESC with miRNA expression levels in the nucleus accumbens (NAc):																
		estimate	0.094	-0.627	-0.087	0.390	0.315	0.203	0.212	-0.011	0.510	0.216	0.076	-0.060	0.744	-0.156	-0.261	0.402
		statistic	0.211	-1.801	-0.196	0.946	0.743	0.464	0.484	-0.024	1.325	0.494	0.170	-0.134	2.487	-0.354	-0.604	0.983
		p-value	0.841	0.132	0.852	0.387	0.491	0.662	0.649	0.982	0.242	0.642	0.872	0.899	0.055	0.738	0.572	0.371

IL-PFC: Infralimbic prefrontal cortex; PL-PFC: Prelimbic prefrontal cortex; NAc: Nucleus accumbens.
Normalized expression levels of miRNAs are listed under the name of each miRNA.
